# Biomechanical effects of periodontal status on molar sequential distalization with clear aligners: a finite element study

**DOI:** 10.1186/s40510-025-00562-6

**Published:** 2025-05-05

**Authors:** Yanning Ma, Xinyue Fan, Xulin Liu, Mingxin Zhang, Zuolin Jin, Jie Gao

**Affiliations:** 1 State Key Laboratory of Oral & Maxillofacial Reconstruction and Regeneration, National Clinical Research Center for Oral Diseases, Shaanxi Clinical Research Center for Oral Diseases, Department of Orthodontics, School of Stomatology, The Fourth Military Medical University, Xi’an, China; 2 Shanxi Medical University School and Hospital of Stomatology, Taiyuan, China

**Keywords:** Molar distalization, Clear aligner, Periodontal disease, Finite element analysis

## Abstract

**Objective:**

Molar sequential distalization with clear aligners was advantageous. However, the effect of periodontal status on it has yet to be investigated. This study aimed to analyze the influence of the different periodontal states on molar distalization to reduce the adverse mechanical stimulation caused by periodontal states by the different stagings of movement and further explore therapeutic recommendations for clinical practice.

**Methods:**

To ascertain the initial displacement of dentition and periodontal ligament (PDL) hydrostatic stress, finite element models (FEMs) were developed. These models included the distalization of the second molars (Step A) and the first molar (Step B) in three distinct periodontal conditions (simulating the periodontal state of mild, moderate, and severe periodontitis) and three distinct distances (0.10 mm, 0.18 mm, 0.25 mm).

**Results:**

Periodontal status affected the tooth movement during molar distalization. During the molar distalization with 0.25 mm step distance, the initial displacement of the molar was greater in the model with worse periodontal condition. However, it did not increase the efficiency of tooth movement because the initial displacement is accompanied by tipping. Moreover, the second molar relapse to mesialization for a reaction from the first molar distalization affected efficiency. Fortunately, reducing the step distance could control those undesired tooth movements positively associated with alveolar bone resorption.

**Limitations:**

The finite element method cannot simulate complex periodontal conditions in clinical practice.

**Conclusion:**

To reduce the undesired tipping and relapse, the personalized staging of movement should be designed according to the periodontal condition. Designing 0.18 mm step distance for patients with 1/3 alveolar bone resorption is recommended, whereas patients with 1/2 alveolar bone resorption need 0.1 mm. These recommendations can guide orthodontists in designing effective treatment plans for patients with varying degrees of periodontal disease.

## Introduction

With the increasing number of adult orthodontic patients, orthodontists must pay attention to the periodontal issues of their patients [1]. Periodontal status affects how force is applied during orthodontic treatment [2]. As a new type of orthodontic treatment, clear aligners (CAs) have been accepted by more adult patients due to their aesthetic characteristics. Clear aligners are thermoplastic appliances designed to fit around the unique shape of the crown of the teeth. They exert a restoring force on the teeth through elastic deformation, gradually moving the tooth to its desired position within a small area [3, 4]. Smaller movement and gentler correction appear more suitable for periodontal tissue reconstruction in periodontal patients.

Considering the removable nature of CAs, patients can remove them and thoroughly clean the teeth and appliances to control plaque and maintain oral hygiene [5]. This seems to be advantageous for patients with periodontal disease. However, the center of resistance of the tooth shifts towards the root due to attachment loss and alveolar bone resorption in patients with periodontitis [6]. This disrupts the normal relationship between force and moment, which impacts tooth movements [7].

Moreover, CAs are considered to exhibit excellent efficiency in molar distalization [8]. Compared with the traditional appliance, CA has antagonism on the buccal and lingual sides and does not easily occur the undesired inclination and rotation [9, 10]. Consequently, in the molar distalization, CAs can reduce the extrusion of maxillary first molars and improve the control of occlusal vertical dimension [9]. However, changes in periodontal status affect the distribution of force and moment. It has not been reported whether CAs can still play an advantage in molar distalization in different periodontal conditions.

This study aims to establish a finite element model of molar distalization with a CA in different periodontal conditions and to explore its biomechanical mechanism with different experiment settings. Our purpose was to provide recommendations for treatment design, such as patterns of teeth movement or force principles.

## Methods

Cone-beam computed tomography (CBCT) scan results were collected from a 24-year-old woman with complete dentition, normal tooth size and morphology, healthy periodontal status, third molar extraction, and Class II occlusion. The scans were performed with a full field of view and a center rotation of 360°. The scanning thickness was 0.15 mm, and 668 horizontal layers were reconstructed. Patients signed informed consent to participate in the study, which was approved by the Ethics Committee of The Air Force Medical University (IRBREV-2022079).

For threshold segmentation, CBCT scans were imported into Mimics 20.0 Software (Materialise Software, Leuven, Belgium). The original three-dimensional (3D) model was reconstructed using the Calculate 3D command, and the model was optimized by importing GeomagicStudio12.0 to improve its accuracy and smoothness.

The outer surface of the tooth root was extended by 0.25 mm to create a periodontal ligament (PDL). To simulate articular cartilage, a 0.41 mm cartilage layer was made on the articular surface of the mandibular condyle and temporal bone. All premolars and lower canines were designed with vertical rectangular attachments (2 × 3 × 1 mm ^3^) on the buccal surface, and maxillary second molars were designed with horizontal rectangular attachments (3 × 2 × 1 mm ^3^). Then, CAs are simulated by extending the crowns and attachments outwards by 0.5 mm.

The linear viscoelastic model of the disc is simulated using the generalized Maxwell model through the optimized Prony series [11]. Other materials are simplified as homogeneous, continuous, and isotropic linear elastomers. Table 1 depicts the material parameters [11–14] involved in the experiment.

**Table 1:** Properties of the various materials considered in this study

Material	Young’s modulus (MPa)	Poisson’s ratio
Attachments [13, 14]	1.25 × 10^4^	0.36
Tooth [12–14]	1.96 × 10^4^	0.3
Alveolar bone [12]	2 × 10^3^	0.3
Articular surfaces [11]	7.9 × 10^− 1^	0.49
Clear Aligner [13]	5.28 × 10^2^	0.36
PDL [12, 13]	6.9 × 10^− 1^	0.49
Buttons [13]	1.14 × 10^5^	0.35
Articular disc [11, 12]	1.8 × 10^− 1^	0.4

**Fig. 1:**
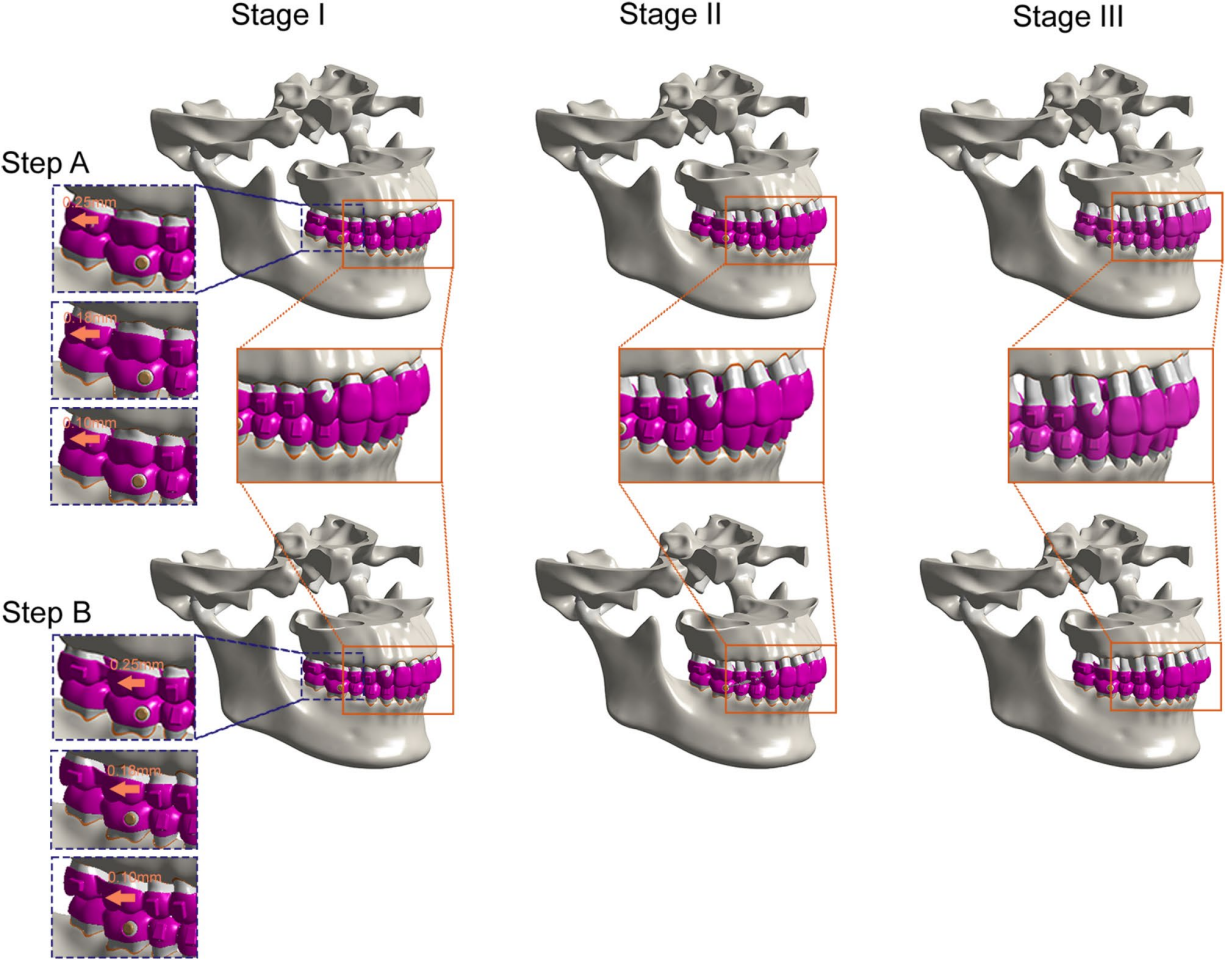
The process of molar distalization under different periodontal conditions was showed when the step distance was set to 0.25 mm, 0.18 mm and 0.10 mm, respectively. Step A: distalization of the upper second molar. Step B: distalization of the upper first molar. Stage I, mild periodontitis without alveolar bone loss; Stage II, moderate periodontitis with alveolar bone loss of 1/3; Stage III, severe periodontitis with alveolar bone loss of 1/2

Figure 1 presents the periodontal status of three groups of three different model designs based on the new classification of periodontal disease (In Stage I, radiological bone loss is in the third of the root length, and the degree of resorption is < 15%; In Stage II, radiological bone loss is in one-third of the root length, and the degree of resorption is 15%~33%; In Stage III, radiological bone loss extending to one-third to two-thirds root length). The process of molar distalization was simulated step by step using the model of different periodontal conditions, such as maxillary second molar distalization (Step A) and maxillary first molar distalization (Step B). The molar distalization was set at a step distance of 0.10 mm, 0.18 mm, and 0.25 mm, respectively.

The models were meshed using tetrahedral elements to ensure accurate representation of the complex geometries. The mesh density was optimized to balance computational efficiency and accuracy, nodes and linear elements of each submodels are shown in Table 2. The contacts between the cortical bone and periodontal ligament (PDL), the PDL and tooth root, and the tooth crown and attachment were securely bonded, preventing mutual movement. Furthermore, the connections between neighboring teeth were not separated from their interfaces, whereas modest quantities of frictionless sliding were allowed along the contact faces. The friction coefficient between the CA and the tooth crown surface and attachments was set to 0.2.

**Table 2:**
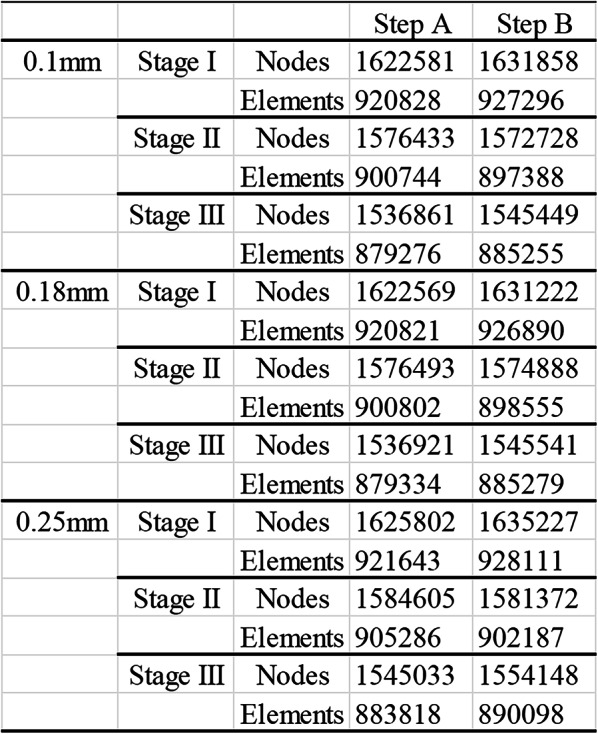
Nodes and elements

The finite element simulation was performed under static loading. In Step A, the loading force was applied by mismatching the initial dentition and the aligner formed by moving the second upper molar, either 0.10 mm, 0.18 mm, or 0.25 mm distally. In Step B, the second upper molars were moved 2 mm distally to the target position. The first molars were then moved 0.10 mm, 0.18 mm, or 0.25 mm distally to form an aligner to exert loading force. Simultaneously, the aligners that form a precision circumcision are combined with Class II elastic traction. The displacement tendency of the whole dentition and individual tooth, the hydrostatic stress on the PDL, and the Von Mises stress of root and alveolar bone were imported by ANSYS Workbench 2019 (Ansys, Pennsylvania, USA).

## Results

**Fig. 2:**
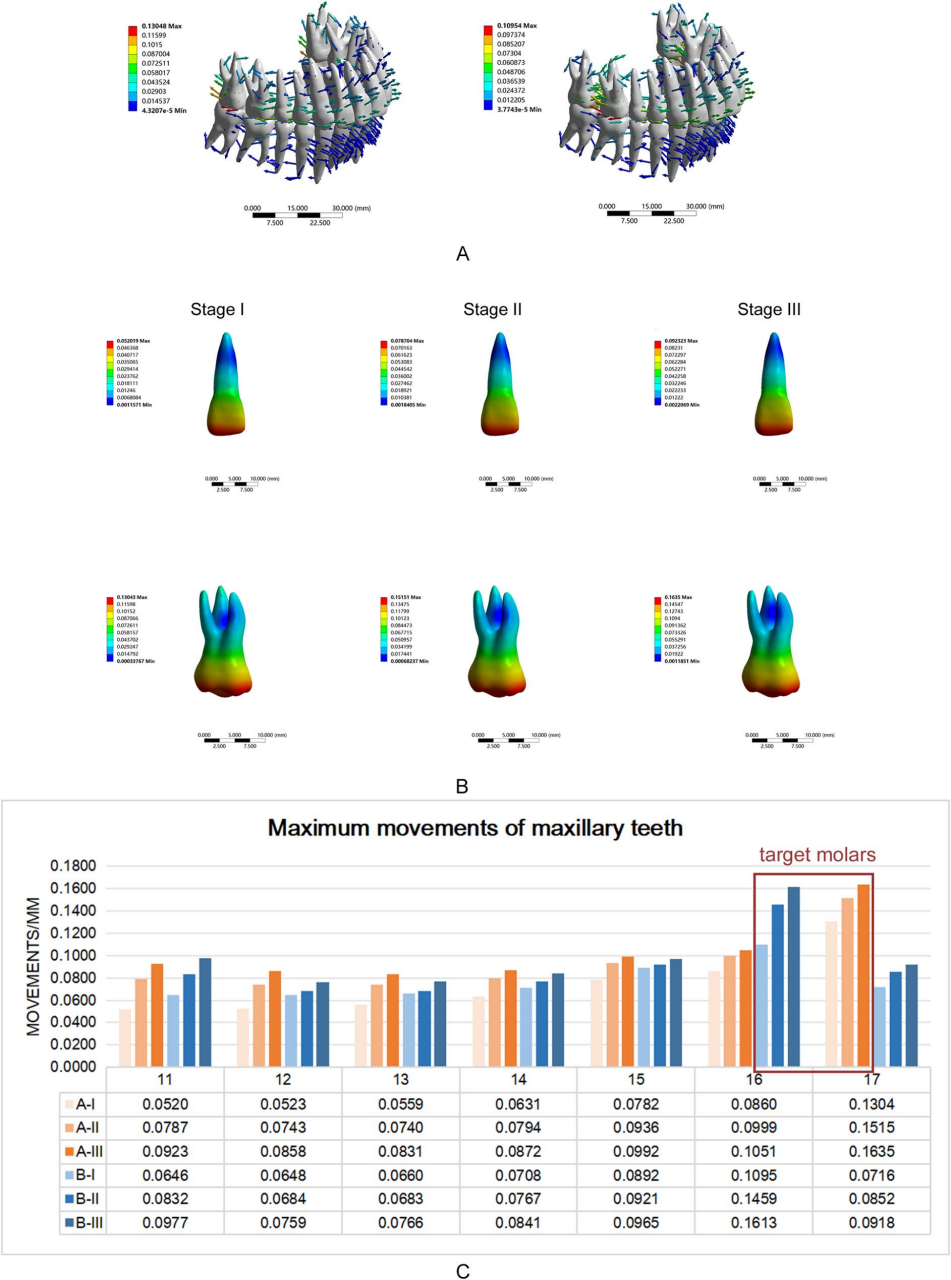
Molar distalization with the step distance of 0.25 mm.** A** Dentition displacement in Stage I without loss of alveolar bone, during molar distalization step by step.** B** The position of the resistance center of the central incisor and maxillary second molar changed under different periodontal conditions when the second molar moved distally (Step A). In the total deformation trend of the central incisor and maxillary second molars, the blue area had the least amount of movement and was considered to be the center of resistance of the tooth.** C** When molar distalization was completed step-by-step, maximum movement of the maxillary teeth were shown in the various stages of the periodontitis model

1. The mechanical performance of teeth under normal periodontal status:

Figure 2A depicts the displacement tendency of the whole dentition in the process of molar distalization with 0.25 mm step distances. The target upper molar crown moved distally, while the other teeth were affected by the opposite force and moved in the opposite direction. Regarding the displacement amount of the crown, the efficiency of the second molar distalization was greater than that of the first molar. Moreover, when the second molar was moved 2 mm distally to the target position, the interaction force generated from the first molar distalization caused the second molars to move mesially. The relapse of the second molar caused an effect of the molar distalization, which was unavoidable.

2. The mechanical performance of teeth under different periodontal status:

Figure 2B depicts the center of resistance gradually moving from the junction of the apical 1/3 and the middle 1/3 of the root to the apical with the periodontal status changes affecting teeth movement. The maximum displacement of the maxillary individual tooth increased during the molar distalization with the same step distance (Fig. 2C).

**Fig. 3:**
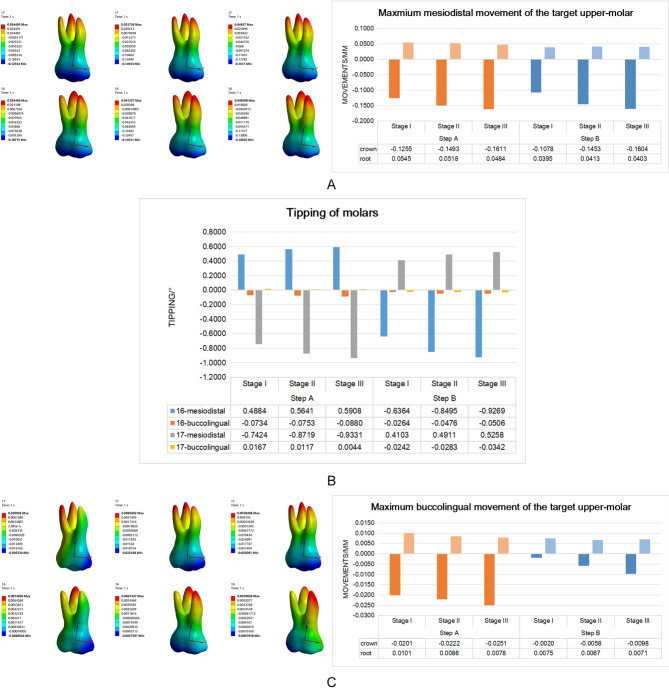
The displacement of target molars in mesiodistal and buccopalatal direction and molars tipping in different periodontitis models with the step distance of 0.25 mm.** A** The mesiodistal displacement of target molars when the molars were removed step by step. The positive value indicated that the root was mesial tipping and the negative value indicated that the crown was distal tipping.** B** The molars tipping were shown in the various stages of the periodontitis model, when molar distalization was completed step-by-step. Positive values: mesial or palatal tipping and negative values: distal or buccal tipping.** C** The buccopalatal displacement of target molars when the molars were removed step by step. The positive value indicated that the root was palatal tipping and the negative value indicated that the crown was buccal tipping

The change of periodontal status resulted in a considerable increase in the initial distal displacement of the second molar crown in Step A and the first molar crown in Step B (Step A: 0.1255 mm, 0.1493 mm, and 0.1611 mm; Step B: 0.1078 mm, 0.1453 mm, and 0.1604 mm) (Fig. 3A). Regarding the displacement amount of the crown, the efficiency of the second molar distalization was greater than that of the first molar. In Stage III, the difference in the initial movement between the first and second molars was narrowed. The increased initial distal displacement was accompanied by a significant increase in the distal tipping (Fig. 3AB). In Step A with 0.25 mm step distance, the distal tipping of the second molar increased (0.74241°, 0.87191°, and 0.93308°); the mesial tipping of the first molar also increased (0.48841°, 0.56405°, and 0.5908°) with the periodontal condition changed (Fig. 3B). The same trend was observed in Step B with decreased alveolar bone height and increased distal tipping of the first molar (0.63635 °, 0.84951°, and 0.92692°). Furthermore, there was an increase in mesial tipping of the second molar (0.41033°, 0.49107°, and 0.52582°).

During molar distalization, the amount of the crown moving to the buccal side increased as the periodontal state changed, and this movement was accompanied by different degrees of buccal inclination (Fig. 3B, C). During the second molar distalization at 0.25 mm step distance, the second molar crown moving to the buccal side increased (0.0201 mm, 0.0222 mm, and 0.0251 mm) from Stage I to Stage III models. The resulting buccal inclination was smaller, possibly because the second molar had more stable anterior anchorage. In Step B, both the amount of the buccal displacement of the first molar crown (0.0020 mm, 0.0058 mm, and 0.0098 mm) and the buccal inclination (0.0264°, 0.0476°, and 0.0506°) increased significantly with the periodontal condition changed.

**Fig. 4:**
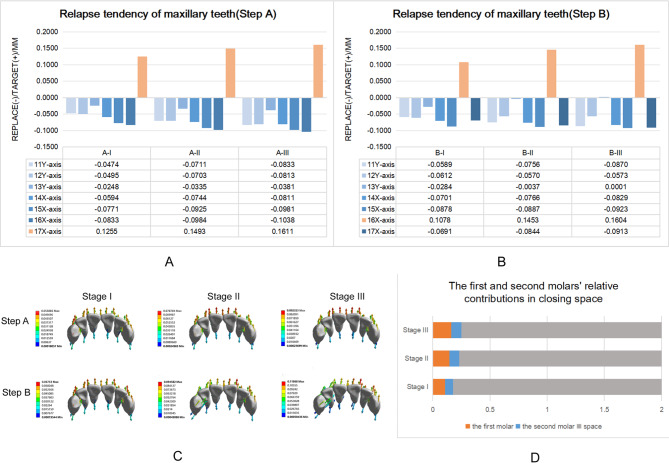
The undesirable displacement of maxillary teeth in the process of molars distalization with 0.25 mm step distance.** A** Step A: The displacement of the second molar distalization and the rest of the teeth moved by reciprocal stress(positive values represented the maxillary second molar moved distally and negative values indicated the remaining maxillary posterior teeth moved mesially and the maxillary anterior teeth moved to labial);** B** step B: The displacement of the first molar distalization and the rest of the teeth moved by reciprocal stress(positive values represented the maxillary first molar moved distally and negative values indicated the remaining maxillary posterior teeth move mesially and the maxillary anterior teeth moved to labial.** C** The movement tendency of the upper anterior teeth as an anchorage unit in different periodontitis models.** D** The bar chart shows the contribution of the first and second molars to the closing of the gap when the first molars are moved(Step B)

We observed the tooth movement in the sagittal direction, where the target molar distalization and the other teeth showed different degrees of mesial or lingual movement, including anterior labial inclination, mesialization of the premolars, and the relapse of the second molar during the first molar distalization (Fig. 4A, B). Figure 4C depicts that as the step distance is set to 0.25 mm, the degree of labial inclination of the maxillary anterior teeth increased with the periodontal condition change, which showed that the distance of the crown moving to the labial side increased. When the second molar was moved distally with 0.25 mm, the displacement of the premolars to the mesial increased with alveolar bone loss (Fig. 4A). Moreover, when the second molar was moved 2 mm distally to the target position, the interaction force generated from the first molar distalization caused the second molars to move mesially. A consequence of the molar distalization was inevitable due to the relapse of the second molar. Notably, the efficiency of the second molar is reduced due to the more obvious relapse that occurs with increased alveolar bone loss (Fig. 4B). During the progress of the first molar distalization with 0.25 mm step distance, the mesialization of the second molar increased (0.0691 mm, 0.0844 mm, and 0.0913 mm) with the periodontal condition changed. When the second molar was displaced distally by 2 mm to reach a target position, the first molar underwent a distal movement of 0.25 mm. Ideally, the 2 mm space between the first and second molars is expected to be filled with the first molar distalization rather than the second molar mesialization occupying this space for interaction force. Figure 4D illustrates the contributions of the first and second molars in closing space in Step B when the step distance was set to 0.25 mm. The teeth movement increased, and the tooth spacing decreased as the periodontal condition changed in Step B. In models with more alveolar bone loss, the teeth moved more efficiently. This was caused by increased undesired movement, such as a more obvious tendency for the second molar relapse to mesialization and an increase in molar tipping.

**Fig. 5:**
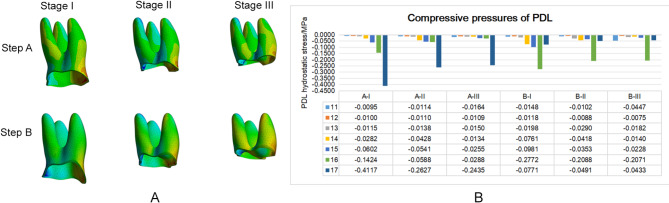
PDL hydrostatic stress was showed under three different periodontal conditions.** A **When the target molar was set to move 0.25 mm, the distribution of PDL hydrostatic pressure in target molars.** B **Bar chart showed the highest compressive stress of the PDL when the target molar was set to move 0.25 mm. Negative values represent compressive pressures

In the maxillary model with a step distance of 0.25 mm, it was found that when the periodontal condition was worse, the maximal compressive stress of the molar PDL tended to decrease (Fig. 5A, B). In Step A, the highest compressive stress of the PDL of the maxillary first molar was − 0.1424 MPa, -0.0588 MPa, -0.0288 MPa; the upper second molar was − 0.4117 MPa, -0.2627 MPa, and − 0.2435 MPa, respectively when alveolar bone is normal, resorption 1/3 or 1/2. In Step B, the highest compressive stress of the PDL of the maxillary first molar was − 0.2772 MPa, -0.2088 MPa, and − 0.2271 MPa; the maxillary second molar was − 0.0771 MPa, -0.0491 MPa, and − 0.0433 MPa, respectively corresponding to Stage I, Stage II, and Stage III.

**Fig. 6:**
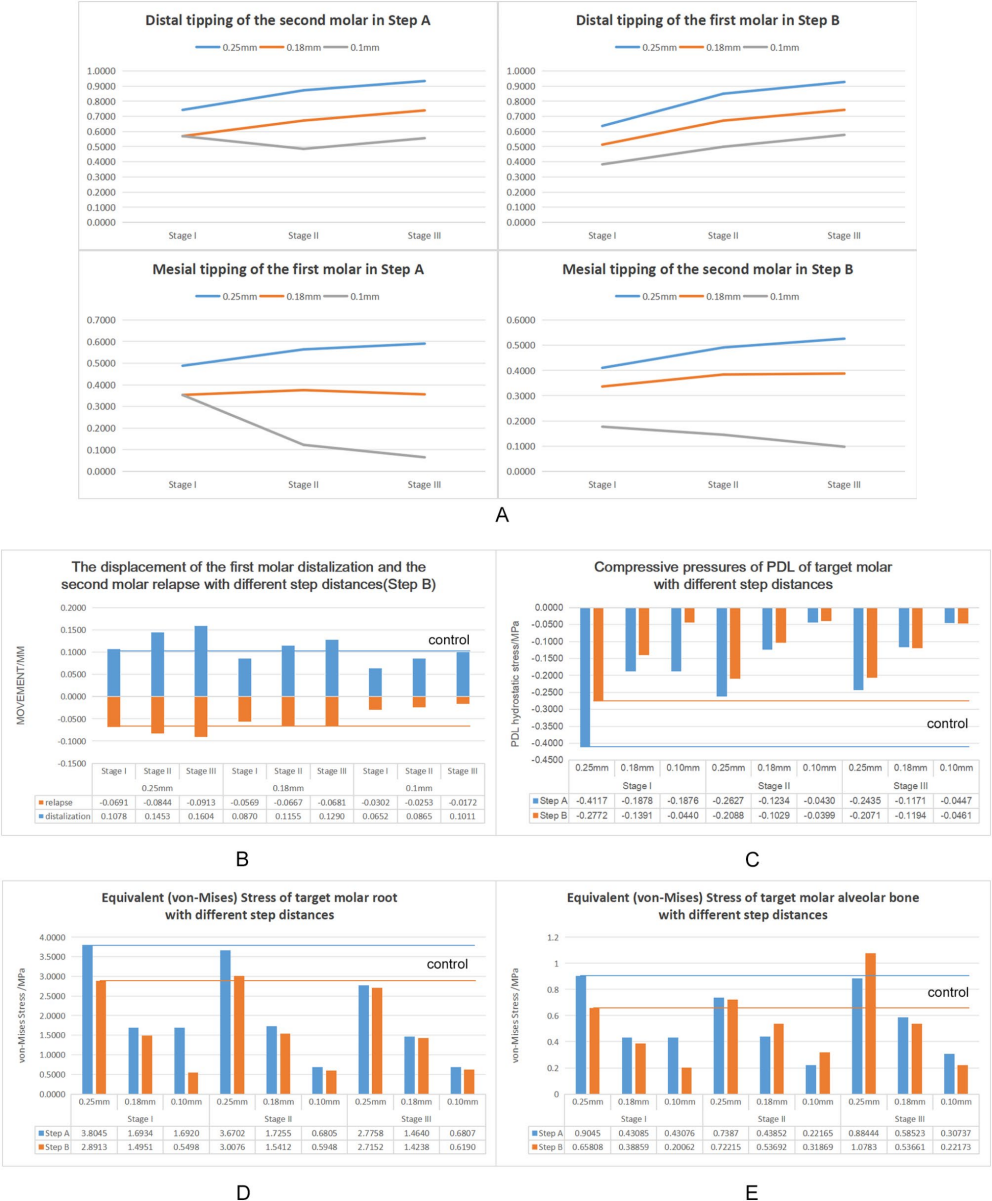
**A** The mesiodistal tipping of molars under the condition of different step distances.** B** The displacement of the first molar distalization and the second molar relapse into mesialization under the condition of different step distances.** C** The highest compressive stress of the PDL for the target molars with different step distances.** D** The highest Equivalent (von-Mises) Stress of the target molars’ roots with different step distances.** E** The highest Equivalent (von-Mises) Stress of the alveolar bone for the target molars with different step distances

3. The mechanical performance of teeth under different steps:

Figure 6A depicts that when the step distance was reduced from 0.25 mm to 0.18 mm and then 0.10 mm, the distal tipping of the second molar (Step A) and the first molar (Step B) in different periodontal conditions showed a downward trend (Step A, Stage I: 0.7424°, 0.5694°, 0.5694°; Stage II: 0.8719°, 0.6722°, and 0.4852°; Stage III: 0.9331°, 0.7391°, and 0.5558°; Step B, Stage I: 0.6364°, 0.5131°, and 0.3830°; Stage II: 0.8495°, 0.6717°, and 0.4990°; Stage III: 0.9269 °,0.7425°, and 0.5775°). Moreover, the mesial tipping of the first molar (Step A)and the second molar (Step B) in different periodontal conditions were also significantly reduced (Step A, Stage I: 0.4884°, 0.3536°, and 0.3535°; Stage II: 0.5641°, 0.3759°, and 0.1229 °; Stage III: 0.5908°, 0.3563°, and 0.0655°; Step B, Stage I: 0.4103°, 0.3365°, and 0.1773°; Stage II: 0.4911°, 0.3840°, and 0.1453°; Stage III: 0.5258°, 0.3878°, and 0.0979°). Notably, excessive reduction in step distance had a limited impact on teeth without alveolar bone defects (Stage I), especially in the second molar distalization at 0.18 mm and 0.10 mm; the distal tipping of the second molar and the mesial tipping of the first molar was not affected by step distance. However, in the alveolar bone defect model (Stage II and Stage III), the distal tipping of the second molar and the mesial tipping of the first molar decreased with the step distance reduction.

Furthermore, when the step distance was reduced from 0.25 mm to 0.18 mm and then 0.1 mm (Fig. 6B), the degree of relapse in the second molar mesialization minimized with the decreasing step distance (Stage I: 0.0691 mm, 0.0844 mm, and 0.0913 mm; Stage II: 0.0569 mm, 0.0667 mm, and 0.0681 mm; Stage III: -0.0302 mm, 0.0253 mm, and 0.0172 mm). Interestingly, when the preset step distance was 0.1 mm, the remission of this relapse was most obvious, reducing the risk of second molar relapse into mesial displacement with more alveolar bone loss.

During the step distance reducing from 0.25 mm to 0.18 mm and then 0.10 mm (Fig. 6CDE), a significant decrease was observed in the periodontal membrane, root and alveolar bone stress in molars. However, in the progress of the second molar distalization with the normal alveolar bone (Stage I), the stress on the root, PDL and alveolar bone did not further decrease when the step distance decreased to 0.10 mm but was similar to the stress at 0.18 mm step distance.

## Discussion

According to the theory proposed by Nägerl et al. (1991), tooth movement based on the assumption of 3D linear elasticity generally implies that in a given plane, the distance from the point of force application to the center of resistance is multiplied by the distance from the center of resistance to the center of rotation to obtain a constant value [15]. Geramy et al. further demonstrated that the Nägerl theory applies to healthy teeth and teeth with alveolar bone loss [16]. Although the product of the distance between the point where force is applied, the center of resistance, and the distance between the center of resistance and the center of rotation remain constant for the tooth with alveolar bone loss and the healthy tooth, the value of this product is smaller in the case of alveolar bone loss [16]. The smaller the value, the smaller the critical zone, making it more sensitive to reach the center of resistance and more difficult to predict the center of resistance [16]. This is the main problem faced by patients with reduced periodontal support during orthodontic treatment. It is often difficult for orthodontists to predict the position of the resistance center, which leads to difficulty in controlling the force value.

Effective tooth movement depends on the relationship between the line of action of force and the center of resistance of a tooth; thus, the location of the tooth center of resistance is critical to predicting tooth movement and minimizing undesirable side effects [17]. The alveolar bone causes a decrease in height and moves the center of resistance of the tooth further toward the apical, increasing the torque of the force [6, 18]. Previous studies have found that the ratio of moment to force increases with the alveolar bone losing [19], which will lead to greater movement and deflection of the teeth and increase unnecessary tipping [7, 20, 21]. This problem is also reflected in our experimental results; as the tooth movement increases, the periodontal condition worsens, and alveolar bone height decreases, which seems to increase the efficiency of tooth movement. However, increased molar distalization is accompanied by increased tipping, which does not improve the efficiency of tooth movement but increases the undesired movement, as we tried to avoid in patients with reduced periodontal support. Undesirable conditions such as extrusion of first premolars, tipping of the maxillary molars, further reduction of alveolar bone height, and the loss of anterior anchorage may occur during the distal movement of the molars [10, 22]. These problems were also reflected in our experimental results; the amount of tooth movement increases as the height of the alveolar bone decreases, which seems to increase the efficiency of tooth movement. However, the increased movement accompanied by increased uncontrolled tipping does not improve the efficiency of tooth movement. Instead, it increases the undesired tooth movement and tipping. Our previous study [23] showed that by moving the tooth with greater tooth inclination, the alveolar bone will be under greater pressure, which is what we try to avoid in patients with periodontal conditions.

In the process of molar distalization, the displacement of the buccal tilting of the molar crown was observed significantly with the increase of alveolar bone loss. Previous study [24] identified that molar distalization increased the width of the dental arch. Unlike the first molar, the periodontal membrane area of the second molar was smaller than that of the first molar, causing its crown to move more (distal and labial). Since the buccal side of the maxillary alveolar bone was relatively thin, the increased movement of the crown toward the buccal side raised the risk of fracture of the buccal bone plate, especially for those with a thinner buccal bone plate. The excessive tipping leads to an increase in the maximum compressive stress at the apical, a condition that may trigger root absorption [19].

In order to protect the periodontal tissue of periodontitis patients, it is also necessary to pay close attention to the stress of the periodontal tissue. We found that when moving the first molar at 0.25 mm, the stress on the root (Stage I: 2.8913 MPa, Stage II: 3.0076 MPa) and alveolar bone (Stage I: 0.6581 MPa, Stage II: 0.7222 MPa, Stage III: 1.0783 MPa) was increased for the deterioration of periodontal condition. In order to avoid the effect of increased stress on periodontal tissue, we set the molar distalization of 0.25 mm in normal alveolar bone as a control group and found that when reducing the step distance to 0.18 mm, the stress of the root (Stage I: 1.4951 MPa, Stage II: 1.5412 MPa) and alveolar bone (Stage I: 0.3886 MPa, Stage II: 0.5369 MPa, Stage III: 0.5366 MPa) could be effectively reduced.

When the periodontal condition is worse, the labial movement of the maxillary anterior teeth increases. Notably, the degree of labial tilting of the maxillary central incisors increases significantly. Considering that patients had varying degrees of loss of periodontal support tissue, the increase in displacement and tipping may increase the incidence of labial gingival recession, bone fenestration, pathological tooth displacement, and other adverse conditions [25, 26]. Increased labial movement of maxillary anterior teeth implies loss of anterior anchorage resistance with increased periodontal tissue loss.From the point of view of displacement amount, the efficiency of the second molar distalization was greater than that of the first molar. The first and second molars’ movement efficiency shrank with the loss of periodontal support tissue. This may be because the root bifurcation angle of the first molars is larger, and the position is relatively more stable at the normal alveolar bone level. As the alveolar bone height decreased to the trifurcation area, the dominance of the root morphology of the first molar disappeared. In the same way, in the case of alveolar bone defect, the second molar mesialization caused by the first molar distalization seriously affects the effect of orthodontic treatment. Fortunately, relapse can be minimized as the step distance reduces. The first molar distalization of 0.25 mm in the normal alveolar bone as a control group, in which the first molar moved 0.1078 mm distally and the second molar moved 0.0691 mm mesially. Because of the loss of alveolar bone, the center of resistance of the tooth is shifted apically, and the tendency of the first molar distalization and the second molar mesial relapse was more pronounced. Fortunately, our results showed that when the step distance was reduced to 0.18 mm, both 1/3 and 1/2 alveolar bone resorption could be considered in guaranteeing the amount of first molar distalization (Stage II: 0.1155 mm, Stage III: 0.1290 mm); Moreover, the relapse of the second molar was controlled (Stage II: -0.0667 mm, Stage III: -0.0681 mm). Therefore, applying a 0.18 mm step distance to move the first molar distally to reduce relapse in patients with different periodontal conditions is more reasonable.

The initial displacement of the molars increases with alveolar bone loss, while reducing the orthodontic displacement may be a safer approach to minimize the undesired tipping movement and protect the remaining periodontal support tissue in patients with periodontitis [23, 27]. Our results verified that reducing the orthodontic displacement facilitates the CA to control the bodily movement of the molars and reduce the uncontrollable tipping caused by the shift of the center of resistance towards the apical. We set the same control group and found that in the progress of the second molar distalization, patients with 1/3 or 1/2 alveolar bone resorption can use 0.18 mm step distance for molar distalization, and the amount of the molars distal tipping is lower than that of the tipping in the control group. Likewise, in the progress of the first molar distalization, patients with 1/3 alveolar bone resorption can achieve tipping reduction with a step distance of 0.18 mm. Notably, Patients with 1/2 alveolar bone resorption may need a step distance of 0.10 mm to control the distal tipping movement of the first molar to less than the control group.

Although our experiments have found patterns of tooth movement under different periodontal conditions, it is worth noting that tooth movement during the active phase of periodontitis is not conducive to alveolar bone remodeling [28, 29]. It is recommended that before orthodontic tooth movement for patients with periodontitis, the orthodontist is expected to first cooperate with the periodontist to evaluate the activity of periodontitis, then apply orthodontic force to move the teeth after the periodontitis is controlled [28, 29]. Simultaneously, patients need to cooperate to maintain oral hygiene and periodontal treatment results for long-term maintenance to avoid the relapse of periodontitis. When periodontal infection has been controlled, applying orthodontic forces increases alveolar bone volume, consequently improving bone quality [30]. It is suggested that combined periodontal-orthodontic treatment may lead to better treatment outcomes for periodontitis patients, such as good periodontal conditions, stable occlusion, and good aesthetic effect [31, 32].

In this study, we explored the biomechanics of CA treatment for patients with different periodontal conditions during upper molar sequential distalization only from the level of mechanics. Yet, in clinical practice, more practical issues, such as oral hygiene and patient compliance, need to be considered. Patients are expected to maintain good oral hygiene and maintain the results of periodontal treatment for a long time to avoid the relapse of periodontitis. When formulating treatment plans for patients with compromised periodontal tissues, it is important to consider the type and severity of periodontal disease. Each patient’s treatment plan should be tailored to their specific needs. Attention should be paid to the characteristics of the alveolar bone of patients with periodontal disease when applying a CA, and appropriate force value or reasonable patterns of tooth movement should be applied to reduce or avoid further periodontal destruction.

## Conclusions

In this study, we presented a finite element simulation of molar distalization with CAs; it was found that alveolar bone loss would lead to greater displacement and tipping of the teeth at the same step distance. Instead of increasing tooth movement efficiency, it increased undesired tooth movement. It is recommended that because of the characteristics of alveolar bone with periodontal disease, an appropriate force value or reasonable patterns of tooth movement should be applied when applying a CA to reduce or avoid further periodontal destruction.

## Data Availability

The data underlying this article will be shared on reasonable request to the corresponding author.
